# Hematopoietic Stem Cell Expansion: Testing the Potential of HOXB4 in Nonhuman Primates

**DOI:** 10.1371/journal.pmed.0030243

**Published:** 2006-05-02

Throughout life, the body's tissues are maintained and repaired by stem cells—self-renewing cells that differentiate into many mature cell types. Every day, for example, the human body makes billions of white blood cells, red blood cells, and platelets from hematopoietic (blood system) stem cells (HSCs) to replace cells lost by normal wear and tear. This process of hematopoiesis helps to maintain a healthy immune system, enables sufficient oxygen to be carried around the body, and ensures effective blood clotting after wounding.

Some people, however, do not have a fully functioning hematopoietic system. They may have been born with a genetic alteration that disrupts the function of some blood cells, or they may have had chemotherapy for cancer that has destroyed their hematopoietic system. One way to help such people, who are often prone to infection, is to provide them with a new supply of HSCs through transplantation. HSCs are found in small numbers in the bone marrow and peripheral blood, as well as cord blood, which is harvested from the umbilical cord at birth. Cord blood is increasingly being used to treat hematopoietic disorders, but the low number of HSCs present in a unit of cord blood means that transplanted cells can be slow to establish themselves (or engraft) in an adult recipient, prolonging the time the patient is susceptible to infections. Consequently, researchers are looking for ways to encourage HSC expansion before transplantation. Xiao-Bing Zhang, Hans-Peter Kiem and colleagues now report that overexpression of a stem cell self-renewal gene called *HOXB4* in HSCs improves their expansion and engraftment in a nonhuman primate model, particularly early after transplantation.

Overexpression of human HOXB4 in mouse HSCs is known to induce their ex vivo expansion without compromising their ability to differentiate. It also encourages the expansion of hematopoietic precursor cells from human cord blood, both in culture and when transplanted into mice. However, because immunodeficient mice do not support the differentiation of all human hematopoietic lineages, they are not an ideal model in which to investigate human HSC transplantation. Zhang, Kiem, and colleagues, therefore, turned to nonhuman primates, a well-established preclinical model for HSC transplantation and gene therapy, to investigate further whether treatment with HOXB4 holds promise for HSC expansion before transplantation.

The researchers first isolated CD34+ cells from six experimental animals—CD34 is a protein that is expressed only by lymphoid and myeloid hematopoietic precursor cells, which together differentiate into all the different blood cells. Next, the researchers split the CD34+ cells from each animal into two batches. One batch was treated with a retrovirus expressing *HOXB4* tagged with a green fluorescent protein marker (HOXB4GFP+); the control batch was treated with a retrovirus expressing a yellow fluorescent protein marker (YFP+). Three or nine to 12 days later, the batches of cells were mixed and transplanted back into their respective donor animals, whose hematopoietic system had in the meantime been destroyed by irradiation. The researchers then tracked the labeled cells as they repopulated the animals.

Zhang, Kiem, and colleagues showed that, in this competitive repopulation assay, *HOXB4* overexpression greatly improved the engraftment of CD34+ cells, particularly when the cells were expanded ex vivo for an additional six to nine days before transplantation. Short-term engraftment (two weeks after transplantation) of HOXB4GFP+ cells was up to 56-fold higher than that of YFP+ cells. Over time, the percentage of HOXB4-expressing cells in the animals' blood declined, but remained higher than the percentage of control cells even after six months, suggesting that *HOXB4* overexpression might also improve long-term engraftment. Finally, the researchers report that three and six months after transplantation both the myeloid and lymphoid hematopoietic lineages contained HOXB4GFP+ and YFP+ cells. HOXB4GFP+ cells were more common in the myeloid lineage than YFP+ cells, but in lymphocytes the pattern was reversed, indicating that *HOXB4* overexpression may have a larger effect on the engraftment and differentiation of myeloid precursors than of lymphoid precursors.

These results suggest that HOXB4-mediated ex vivo expansion of stem cells could be one way to accelerate the engraftment of HSCs from sources that contain limited numbers of stem cells (such as cord blood). Because only small numbers of animals were used in this proof-of-principle study, more experiments will be needed before it is clear whether HOXB4 can be used to improve the expansion and engraftment of CD34+ cells in patients whose hematopoietic system has failed. As the researchers point out, the availability of recombinant HOXB4 protein makes it possible to treat HSCs directly, without the potential problems associated with genetic manipulation of the cells.

## 

**Figure:**
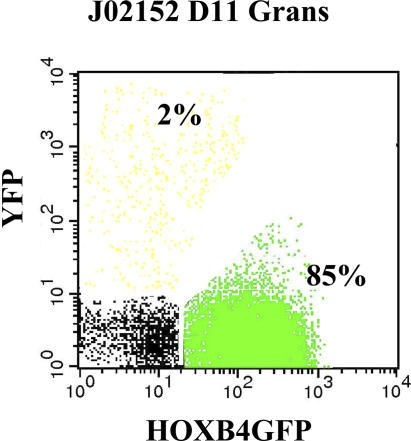
There is a high percentage of HOXB4GFP ^+^ cells early after transplantation

